# How deep is our anxiety during treatment of thyroid cancer?

**DOI:** 10.20945/2359-3997000000051

**Published:** 2018-05-07

**Authors:** Flavio Carneiro Hojaij

**Affiliations:** 1 Universidade de São Paulo Departamento de Cirurgia Faculdade de Medicina Universidade de São Paulo Brasil Departamento de Cirurgia da Faculdade de Medicina da Universidade de São Paulo (FMUSP). Disciplina de Topografia Estrutural Humana da FMUSP. Laboratórios de Investigação Médica (LIM/02) do Hospital das Clínicas da FMUSP

The sentinel lymph node procedure is a technique that aims, with minimal surgical invasion, to diagnose lymph node metastasis and, later, to define the prescribed treatment: full lymph node dissections, radiotherapy, chemotherapy and others. It requires tools such as lymphographies, scintigraphies and surgical approaches followed by anatomopathological and immunohistochemical analyses (1).

Its use began in the 1960s in facial epidermoid carcinomas. In the 1970s, it was incorporated in the treatment of penile carcinomas and, in the last 20 years, it has been used in the treatment of cutaneous melanomas and malignant neoplasms in the mammary exocrine glandular tissue (2).

In Head and Neck Surgery, the sentinel lymph node procedure has gained strength in the last decade, mainly for the treatment of oral cavity tumors; but, due to the learning curve and the complicated logistics, it has not become popular in our midst (1).

The study conducted by Steck and cols., present in this issue of *Archives of Endocrinology and Metabolism* (AE&M) (3), poses legitimate questions about the comprehensiveness of the treatment of well differentiated thyroid carcinoma. The authors state that the treatment should vary according to its need. Should there be a metastasis in a lymph node, the best conduct, according to the authors, is a therapeutic neck dissection.

However, this work arrived in the middle of a hurricane, which is how I’ve been calling the present scenario. We are experiencing a “hurricane” of indagations, directives and controversial information about the aggressivity of well differentiated thyroid carcinomas. The authors of the article even express their anguish about this in their Introduction and Discussion (3,4).

Certainly, the tumors we treat nowadays are smaller than the ones we have treated 20 years ago. These new features are directly influenced by an increase in the number of diagnostics, which is the result of better exams being performed and in a greater amount (4-6).

Faced with so much data and those clinical characteristics, we, the ones who treat the patients, live in an atmosphere of bipolarity. In one hand, we have the active observation suggested for thyroid neoplasms (4,5) but, on the other hand, another authors (3,6-8) stress the danger of lymph node metastases and give us tools with which to handle with them.

Due to this duality, some questions must be raised:

1. Do we really need to be concerned about lymph node metastases that aren’t detected neither in the preoperative ultrasound nor by the surgeon during the procedure?
2. Does the detection of micrometastases (submillimetric) in millimetric lymph nodes play a clinical role and an impact on the survival of patients?
3. Should the sentinel lymph node procedure, which has already been validated, follow the same precepts and logistics established for other tumors in the case of malignant thyroid neoplasms?
4. If so, can it be put into practice?

The answers to these questions will surely be controversial and, considering the polarization we face in oncologic thyroidology, I think the moment demands calm. We should adapt extreme conducts to intermediary positions, opting for partial surgeries instead of limiting ourselves to two radically opposite postures: mere observation or comprhensive surgeries.

We should also individualize treatment, so that its comprehensiveness will be adequate to the patients’ background, culture and expectations. And, finally, we should wait for the development of molecular tools capable of guiding and further helping the daily clinical practice (8,9).
